# Novel nicotine and tobacco products in pediatric age: a joint position paper

**DOI:** 10.1186/s13052-025-02116-2

**Published:** 2025-09-24

**Authors:** Antonio Corsello, Valentina Agnese Ferraro, Laura Reali, Laura Venditto, Mattia Spatuzzo, Maria Elisa Di Cicco, Michele Ghezzi, Luciana Indinnimeo, Stefania La Grutta

**Affiliations:** 1 Department of Clinical-Surgical, Diagnostic and Pediatric Sciences, University of Pavia, Pavia, Italy; 2 Department of Clinical Sciences and Community Health, University of Milan, Milan, Italy; 3 Unit of Pediatric Allergy and Respiratory Medicine, Women”s and Children”s Health Department, University Hospital of Padua, Padua, Italy; 4 European Confederation of Primary Care Pediatricians, Lyon, France; 5 Paediatric Respiratory Unit, Great Ormond Street Hospital for Children NHSFoundation Trust, London, UK; 6 Department of Maternal, Infantile and Urological Sciences, SapienzaUniversity of Rome, Rome, Italy; 7 Section of Pediatrics, Department of Clinical and Experimental Medicine,University of Pisa, Pisa, Italy; 8 Pediatric Department, “Vittore Buzzi” Children Hospital, Milan, Italy; 9 Institute of Translational Pharmacology, National Research Council, Palermo, Italy

**Keywords:** e-cigarettes, ENDS, HTP, Nicotine pouches, Adolescent vaping, Tobacco prevention strategies

## Abstract

Electronic nicotine delivery systems (ENDS), heated tobacco products (HTP), and nicotine pouches have rapidly gained popularity among adolescents, driven by appealing flavors, targeted marketing strategies, and widespread misperceptions of reduced harm. This joint position paper, endorsed by the Italian Society of Pediatrics (SIP) and the Italian Pediatric Respiratory Society (SIMRI), considers current evidence on patterns of youth use and outlines potential prevention strategies. We examine industry tactics, including social-media influencer campaigns and product design features that disproportionately attract adolescents, and discuss the influence of peer, family, and environmental factors on product uptake. Parents and caregivers play a pivotal role through open dialogue, modeling nicotine-free behaviors, and monitoring access. Pediatricians and primary-care providers should incorporate routine screening for all nicotine products into well-child visits, deliver brief motivational counseling, and connect families with cessation resources tailored to teens. Continuous surveillance of youth consumption patterns and systematic evaluation of intervention effectiveness will ensure strategies remain responsive to evolving product designs and marketing practices. Through coordinated policy changes, healthcare support, community action, and education, it is possible to prevent nicotine initiation among adolescents and foster a generation free from smoke and vaping addiction.

## Introduction

The rapid emergence of novel nicotine and tobacco products has created a paradigm shift in tobacco consumption worldwide. In recent years, the diffusion of Electronic Nicotine Delivery Systems (ENDS) such as electronic cigarettes (e-cigarettes), Heated Tobacco Products (HTPs), and nicotine pouches, has challenged regulations and posed new risks, particularly for children [1]. Global data indicate that more than 37 million adolescents aged 13 to 15 currently use novel nicotine and tobacco products, with increasing and extensive marketing sponsored by the industry [2]. A recent WHO report estimates that advertisements for e-cigarettes, nicotine pouches, and HTPs have been viewed over 3.4 billion times on social media platforms [3–5].

ENDS deliver aerosols containing nicotine and various chemicals by heating liquids or tobacco leaves, thereby bypassing combustion. Studies highlight health risks of ENDS use, including nicotine addiction and potential harm to oral, cardiovascular, respiratory, and mental health, as well as adverse pregnancy outcomes [6–8]. Concerns have also been raised regarding long-term health effects, given the oxidative stress, inflammatory responses, and cancer risk associated with exposure to heavy metal and chemicals such as nitrosamines, formaldehyde, and acrolein generated during heating [9–11]. Another significant issue is the dual use of ENDS and HTPs alongside conventional cigarettes, which further increases health risks [12, 13]. Additionally, aerosols pose risks also to bystanders through secondhand exposure and potentially through thirdhand exposure [8, 14]. While reduction strategies for current adult users remain important, preventive measures to protect children and adolescents from early nicotine exposure represent urgent needs. When discarded into soil and water, e-cigarette butts and filters, composed of synthetic plastics, release hazardous chemicals before transforming into microplastic pollution. Discarded e-cigarette butts pose a serious hazard to human health, wildlife, and the environment, an issue that requires targeted policy interventions [15, 16].

Data from the 2024 National Youth Tobacco Survey among U.S. middle and high school students show that in 2024, more than 10% of high school students and 5.4% of middle school students reported current use of at least one tobacco product [17]. E-cigarettes were the most commonly used products (5.9%), followed by nicotine pouches (1.8%), cigarettes (1.4%), cigars (1.2%), smokeless tobacco (1.2%), and other oral nicotine products (1.2%). Most youth vapers use flavored products, with over 80% starting with them, and more than one in four report daily use [18, 19]. The most recent update on adolescent tobacco use in Europe comes from the 2024 European School Survey Project on Alcohol and Other Drugs (ESPAD) [20], conducted across 37 countries, which highlights emerging trends and raises new concerns. ESPAD data show that e-cigarette use has increased dramatically among adolescents, along with rising rates of early initiation and daily use. Dual use of traditional cigarettes and e-cigarettes is also rising, underscoring a broader shift toward alternative nicotine products.

Smoking remains more prevalent among girls in more than half of the ESPAD countries. Notably, about 3.6% of students who began smoking at or before the age of 13 report daily use. Approximately 44% of students report having used e‑cigarettes at least once, and 16% report early e-cigarette initiation at the age of 13 or younger.

In Italy, the data are equally alarming. According to the Global Youth Tobacco Survey conducted in 2022, the use of e-cigarettes among current smokers increased over four years from 18% in 2018 to 20% in 2022. This reflects a decline among boys (from 22 to 18%) but a rise among girls (from 13 to 21%) among students aged 13–15 years old in Italian schools. To note, 15% of students reported being able to purchase products directly from retailers despite the legal ban [21]. These findings highlight the need for a rigorous application of existing restrictions, which would otherwise be in vain.

This joint position paper, endorsed by the Italian Society of Pediatrics (SIP) and the Italian Pediatric Respiratory Society (SIMRI), aims to recommend prevention and intervention strategies, including regulatory and educational actions. In addition, it evaluates youth perceptions and industry influences that sustain this trend and also seeks to support policy-making efforts while raising public awareness.

## Methods

Panel members, nominated by the two endorsing scientific societies, declared no potential conflicts of interest. The final document was reviewed and approved by both societies prior to submission. A targeted literature review was undertaken to identify sources pertinent to youth exposure and outcomes. Eligible evidence included original studies, surveillance reports, systematic reviews, meta‑analyses, clinical guidelines, and policy or technical documents from recognized public health agencies. Studies were considered if they reported on prevalence, risk perceptions and access, prevention and cessation interventions, or regulatory measures related to children, families and caregivers.

Searches were conducted in PubMed/MEDLINE and complemented by targeted queries of authoritative websites (e.g., World Health Organization, European and national surveillance platforms, National Youth Tobacco Survey). Core keyword strings combined product terms (“e‑cigarette,” “vaping,” “heated tobacco,” “HTP,” “nicotine pouch,” “oral nicotine”) with population terms (“adolescent,” “youth,” “pediatric”) and outcome or policy terms (“prevalence,” “epidemiology,” “trends,” “cessation,” “prevention,” “policy,” “regulation,” “marketing,” “flavor,” “packaging,” “harm”). Titles and abstracts were screened to assess relevance, followed by full‑text review for inclusion.

This publication is a position paper, based on a targeted narrative review and expert consensus, intended to guide clinical practice and policy rather than to serve as a systematic review. Consequently, formal risk-of-bias tools were not systematically applied, and priority was given to evidence derived from national and international surveillance datasets. Searches may have missed relevant studies outside the predefined timeframe or languages. Regulatory contexts are dynamic and may have changed after the time of writing.

## Youth perceptions and industry influence

Surveys indicate that approximately half of adolescents perceive e-cigarettes and HTPs as less harmful than cigarettes [22]. These misconceptions are particularly prevalent among older teenagers and males, and are strongly associated with personal use, household use, and advertising exposure. In a large Polish survey of over 12,000 teens, 52.2% believed e-cigarettes were less harmful, and 61.9% thought HTPs were less harmful, compared to traditional cigarettes [22]. In the 2025 ASH Smokefree Great Britain Youth Survey on vaping, 3044 young people were interviewed, including 2746 aged 11–17. Perceptions of harm from vaping are rising, with 63% of respondents now believing that vaping is as harmful as, or even more harmful than, smoking [23]. In 2013, 73% of young people believed that vaping was less harmful than smoking. These misperceptions were more common among older teens, males, those with personal or family use of nicotine products, and those exposed to vaping [24].

Importantly, appealing product design, sweet or fruity flavors, and targeted marketing strategies (such as social media promotion and influencer endorsements) make these products especially attractive to youth. Beyond flavors, industries deliberately exploit aesthetic features to enhance appeal: bright colors, sleek or tech-like designs, compact formats resembling cosmetic items, and accessories that mimic lifestyle products rather than tobacco. Such strategies, particularly effective among young women, extend the market reach of ENDS by embedding them in youth culture and identity, amplifying curiosity and experimentation. Flavors are often cited among the primary reasons why teenagers initiate ENDS use [4]. Thus, the vaping industry’s tactics (including social media hype and flavorings) foster positive perceptions of vaping among adolescents [5]. In addition, sales of nicotine pouches increased fivefold in less than three years, with new marketing authorizations granted every year [25, 26]. Although these novel products are often promoted as less harmful alternatives to conventional cigarettes for adult smokers, their rising popularity among adolescents is cause for serious concern.

The controversial promotion of ENDS as a smoking cessation tool has also contributed to their widespread use among adult smokers. This perception has also led many to consider ENDS as less dangerous, resulting in more permissive use in settings where smoking is otherwise avoided (for example, at home) [27].

Peer influence and parental behaviors also contribute to adolescents’ perceptions and use of these products and other substances [28]. Teens are more likely to experiment with e-cigarettes if they have friends or family members who use them [5]. Moreover, parental smoking or vaping can inadvertently signal to adolescents that such behaviors are acceptable, further diminishing perceived risks [29].

Furthermore, regarding the role of parents and siblings in the use of nicotine and their dependence, they are considered predictors of regular cigarette smoking and ENDS use in adolescents [30]. Findings from the PATH (Population Assessment of Tobacco and Health) Study of 12- to 17-year-olds reported that exposure to secondhand smoke, tobacco use in the home, and peers’ ENDS use are strong predictors of initiation, confirming that peer influence remains a key driver of nicotine uptake over time [31].

Data from the ESPAD report show that more than half of students (55%) consider cigarettes fairly or very easy to obtain. Boys are more likely than girls to perceive cigarettes as easily accessible (61% versus 50%) [31]. Overall, perceptions of reduced harm and the less restricted regulations for e-cigarettes, HTPs, and nicotine products make them highly attractive alternatives to traditional cigarettes [32]. Policymakers need to consider the perceived appeal and reduced harmfulness of these products as important determinants to consider when designing health protection policies.

## Prevention, intervention, and community strategies

### Parents and caregivers

Addressing the rising use of novel nicotine and tobacco products among adolescents requires comprehensive prevention strategies. Evidence suggests that no single approach is sufficient; rather, layered strategies in policy, education, and healthcare are needed. Educational campaigns that provide accurate information about the risks associated with e-cigarettes and heated tobacco products are essential. Parental involvement also plays a critical role: adolescents who receive strong support and clear communication from parents about the dangers of vaping are less likely to initiate such behaviors [33, 34]. Moreover, parents often underestimate the harmfulness of passive exposure to aerosols, which contain fine particles, heavy metals, and other volatile organic compounds. Educational intervention should highlight that second-hand exposure to ENDS and HTPs may negatively affect respiratory health and neurodevelopment in children, reinforcing the need for smoke-free and vape-free homes. Counseling efforts should suggest that families recognize early signs of tobacco/nicotine use and encourage open dialogue at home.

### Schools

School-based interventions have shown promise in increasing awareness and reducing intentions to use these products, even with uncertain impact on actual vaping behavior in the long term [35].

Currently, there are few trials which assessed interventions to prevent or reduce e-cigarette use in children and adolescents [36]. A two-armed randomized controlled trial conducted in Denmark among more than 2,000 students analyzed the effectiveness of a comprehensive school tobacco policy in preventing the transition to poly-tobacco use [37]. Students in the intervention group demonstrated 36% lower odds of transitioning from cigarette use at baseline to poly-tobacco use at follow-up compared with peers in control schools. This policy has been further supported by an online tobacco prevention program, which reduced both the intention and willingness to vape or smoke cigarettes among students, particularly for those at high risk. These findings highlight the effectiveness of school policies that can be implemented by lawmakers.

Integrating cessation programs within schools and community centers provides accessible support for youth. Programs that combine behavioral education with peer support about the risks of nicotine could be effective in reducing its use. Additionally, trauma-informed interventions that address underlying factors contributing to substance use may help in preventing initiation among high-risk adolescents [38, 39].​

### Clinicians

**Fig. 1:**
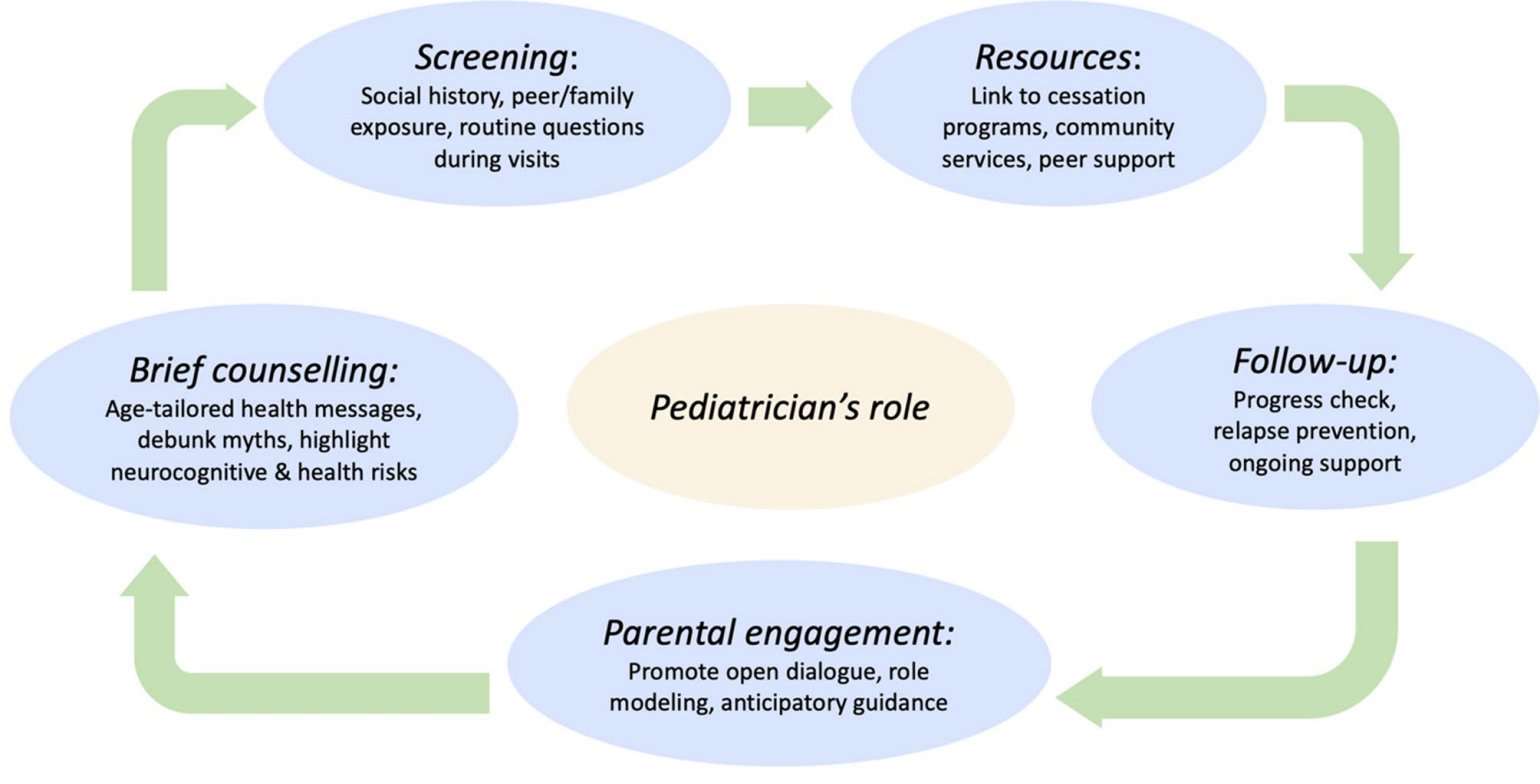
The role of pediatricians in the prevention of nicotine and tobacco use among children and adolescents

Pediatricians and primary care providers occupy a critical position in the prevention of e-cigarette use and nicotine addiction among children and adolescents (Fig. 1) [19, 40]. Their role includes the routine integration of screening, health education, preventive counseling, and appropriate referrals within the clinical care of youth populations. Furthermore, ongoing training and professional development, through dedicated workshops, webinars, and inclusion of vaping-related content in pediatric curricula, are essential to ensure that providers remain confident and up-to-date in addressing novel nicotine products effectively. Standard clinical visits present a key opportunity for intervention. Healthcare professionals are strongly encouraged to implement systematic screening for all novel products, including e-cigarettes, HTPs and nicotine pouches. Such screening should involve developmentally appropriate, direct questioning to assess both current use and prior experimentation with nicotine-containing products. Beyond identification, clinicians should deliver counseling interventions that are both preventive and supportive. These include providing evidence-based education on the substantial health risks associated with nicotine exposure during adolescence, including its detrimental effects on neurodevelopment, cognitive performance, addiction potential, and its role as a possible gateway to other substances [41]. Providers (including pediatricians and teachers) should communicate that e-cigarettes are not a safe alternative to combustible tobacco products and that they contain toxic compounds such as heavy metals, volatile organic compounds, and chemicals.

Counseling efforts should extend to parents and caregivers, who strongly influence adolescents’ health behaviors. Providers should suggest that families recognize early signs of tobacco/nicotine use, encourage open dialogue at home, and foster supportive environments that decrease the likelihood of initiation. For instance, during well-child visits, using targeted anticipatory guidance, including digital ones, following public health authorities’ guidelines. All clinicians play a critical preventive role by routinely asking about any tobacco or nicotine use, including e‑cigarettes and pouches, during well visits and documenting it as part of the social history. Education should emphasize that no tobacco product is safe for young people, that even brief vaping can lead to dependence, and that vapors often contain harmful chemicals. When use is identified, clinicians must offer brief motivational counselling, refer patients and their families to cessation resources, and monitor progress at follow‑up.

Importantly, brief, tailored health messages delivered by trusted healthcare professionals have been shown to influence adolescent behavior significantly. Given the high level of trust typically afforded to pediatricians and family physicians, their guidance can reinforce anti-vaping norms and strengthen preventive messaging both within both clinical and home settings [42]. Effective approaches may include behavioral counseling, motivational interviewing, or engagement with school- or community-based tobacco cessation programs specifically tailored for youth.

### Social media and community strategies

Studies conducted on young adults have explored the possibility of using social media for vaping prevention, and this approach has also appeared effective in recent trials to quit vaping and prevention [43–45]. In fact, given the importance and the massive use of social media by children and adolescents, a study has evaluated how young adults perceive Instagram e-cigarette education posts by three different source types: a health expert, friend, and influencer, interestingly the posts published by the experts had higher perceived source credibility, trust, and curiosity, suggesting the involvement of health experts to improve the delivery and effectiveness of e-cigarette education messages on social media [46]. Finally, when tobacco or nicotine use is identified, pediatricians and primary care providers should be prepared to deliver or refer patients to appropriate, evidence-based cessation interventions (Fig. 1).

## Regulatory frameworks

In the United States, the federal “Tobacco 21” law (2019, strengthened in 2024) forbids the sale of all tobacco and nicotine products, including e-cigarettes, HTPs, and nicotine pouches, to individuals under the age of 21. E-cigarettes and heated tobacco products are regulated under Tobacco 21 but are not approved as smoking cessation aids [47]. In August 2024, the Food and Drug Administration (FDA) further strengthened these regulations, focusing on unlawful items and sales to minors, especially flavored e-cigarettes and nicotine pouches [48]. Supplementary state and local regulations include taxation (Illinois), flavor bans (New York), restrictions on online sales (Vermont), and “Nicotine-Free Generation” statutes (Massachusetts). The FDA is currently proposing a tobacco product standard to regulate nicotine yield in cigarettes and other combusted tobacco products by September 15, 2025. This initiative aims to reduce addictiveness and support individuals in their efforts to quit smoking [47].

Australia strictly regulates nicotine containers and e-cigarettes, with vaping products exclusively available at pharmacies for smoking cessation purposes [49]. Nicotine pouches are unlawful without prescription and have not been approved for safety and efficacy [50]. New Zealand regulates e-cigarettes, HTPs, and nicotine pouches, focusing on flavors, nicotine levels, and age of sale to reduce appeal to young people. The Act includes restrictions on flavors, nicotine levels, and age of sale, with the aim of reducing the appeal of these products, especially among young people [51]. The retail sale of nicotine pouches is prohibited without specific medical approval.

In the United Kingdom, the Tobacco and Related Products Regulations regulate e-cigarettes by establishing restrictions on nicotine concentration, refill volumes, packaging, labeling, and advertising [52]. HTP are subject to the generic tobacco framework, which lacks any specific provisions. In contrast, nicotine pouches are currently regulated solely as general consumer products, with no restrictions on the age of sale, nicotine content, flavors, or marketing. Since June 1st, 2025, a ban on disposable vapes has come into force as the British government aims to stem their use and reduce health risks and litter, preventing the leaking of harmful chemicals into the environment. It is estimated that up to 5 million disposable vapes are discarded each week in the United Kingdom rather than being recycled [53]. In February 2025, France proposed to EU Commission to ban also nicotine pouches, classifying them as poisonous substances. (2025). The European Commission issued this notification, but its implementation was suspended until August 2025 following formal objections from several EU member states [54].

Germany regulates e-cigarettes and refill containers, with sales to minors under 18 prohibited under the Protection of Young Persons Act, which explicitly covers e-cigarettes and refill containers, with penalties for violations. HTPs are treated as conventional tobacco products [55]. Nicotine pouches are classified as unauthorized “novel foods” and commercial sale is prohibited unless specifically authorized under EU novel food regulation.

The regulation of e-cigarettes poses a significant challenge for global health authorities due to the rapid evolution of ENDS and their growing popularity, particularly among youth. Initially introduced as smoking cessation aids, e-cigarettes have evolved into recreational products, often marketed through channels attractive to adolescents [56]. Regulatory efforts vary widely across countries. Formally, all countries ban sales of nicotine products to minors, but enforcement varies. In most of the EU, legislation remains relatively lenient, indirectly, while in Australia, stricter controls have been implemented, allowing ENDS sale only via prescription. The U.S. has raised the minimum age for purchase to 21 years [57, 58]. In particular, the Australian policy seems quite effective in reducing vaping among youth. A comparative study between found that although New Zealand’s less restrictive policies, contributed to faster declines in adult smoking, they may also have increased youth vaping [59].

Despite such efforts, regulatory loopholes persist. For instance, nicotine-free vapes remain accessible to minors, and social media marketing continues to evade age-gating protocols. Many regulations impose product safety standards but do not formally approve e-cigarettes as smoking cessation tools [60, 61]. Also, the online market usually has weak control and less strict regulations, allowing people to escape from the bans. For instance, a study conducted in California [62] showed a rise in online shopping queries following the introduction of flavor restriction, showing that on-line market rules must be enforced in order to prevent irregular selling.

In Italy, the regulation of e-cigarettes falls under a combination of national and European Union laws. The main primary legislative framework comes from the European Tobacco Products Directive 2014/40, which Italy has implemented in 2016 [63, 64]. Italy regulates e-cigarettes as tobacco products, prohibiting sales to individuals aged 18 and over, banning online and distance sales, and restricting use in government buildings [65]. HTPs have lighter regulatory requirements and preferential taxation. Nicotine pouches, initially unregulated, have been subject to control since 2022, but are not covered by the tobacco regulations. This law regulates the manufacture, presentation, and sale of ENDS and refill containers. E-cigarette products must comply with rules on ingredient disclosure, health warnings, packaging, and advertising restrictions. Nicotine-containing e-liquids must not exceed a concentration of 20 mg/mL, and their packaging must include child-resistant mechanisms and warnings about addiction [66]. Italy prohibits the sale of e-cigarettes and HTPs to individuals under 18 years of age. Retailers must verify age both in physical stores and online, although enforcement of these rules has been inconsistently applied [67]. Moreover, in Italy, advertising is tightly restricted: promotional content for e-cigarettes is banned on TV, radio, newspapers, and magazines. However, on social media, youth-targeted content may still appear. To counteract this, the Italian Ministry of Health periodically issues public health warnings and campaigns highlighting the risks of youth vaping and nicotine addiction. Italy also imposes a tax on e-liquids, both with and without nicotine. This tax was initially high, leading to the growth of an illicit market and consumer pushback. In response, the government revised the tax structure in 2019 to make legal products more accessible while maintaining revenue and public health goals. Nonetheless, in 2022, 15% of 13-to 15-year-olds reported buying e-cigarettes directly from retailers, with nearly half getting them from a relative or friend. Furthermore, according to the Global Youth Tobacco Survey, more than 70% of current vapers who attempted to buy e-cigarettes or HTPs from tobacconists reported not being refused by the retailer because they were underage [21].

United Kingdom is another example that legislative intervention can actually determine a fast response in tackling vaping addiction. In 2024, the British government announced plans to introduce new vaping policies, including a ban on disposable vapes, in order to prevent youth vaping [68]. A recent study which uses data from the Smoking Toolkit Study, an ongoing monthly cross-sectional survey on young people from 16 years of age, the prevalence of young “vapers” stabilized rather than increasing [69]. With the disposable vape ban introduced in June 2025, its long-term impact on future generations will warrant close monitoring [53]. In the US, seven states have policies restricting the sale of flavored e-cigarettes. A recent cross-sectional study has shown some reduction in e-cigarette use among young adults but has caused an unintended increase in traditional cigarettes consumption [70]. This shows that one policy is not sufficient, and multiple legislations are needed to prevent youth vaping. Table 1 summarizes an overview of different regulations on ENDS.

**Table 1:** A comparative overview of different regulations

Country	E-cigarettes / ENDS	HTPs	Nicotine pouches	Min. age / ID check	Online sales	Key 2024–25 updates
USA	Tobacco 21 (21+); FDA rules on packaging, warnings	IQOS authorized under PMTA; not as cessation aids	Covered by Tobacco 21; FDA enforcement on youth sales (e.g., ZYN)	21+; ID mandatory if < 30	Online sales heavily restricted (PACT Act)	2024: stronger ID rules and vending ban; FDA crackdowns
United Kingdom	TRPR 2016 (≤ 20 mg/mL nicotine, MHRA notification, warnings)	Treated as tobacco; promotion restricted	Currently consumer products will fall under the Tobacco and Vapes Bill.	18+; retailer duty	Allowed but regulated	Ban on disposables (June 2025); Tobacco and Vapes Bill in Parliament
Australia	Vapes only sold via pharmacies; therapeutic-only since 1 July 2024	HTPs controlled under customs import rules	Only legal with prescription (Therapeutic Goods Act)	18+	Online import requires prescription or scheme	2024: pharmacy-only model; strong restrictions
New Zealand	Smokefree Act, prohibits sale to born after 1 Jan 2009; 18 + otherwise	Same framework; risk-proportionate plan pending	Oral nicotine products illegal retail; personal import only	18+; generational ban applies	Notification to Ministry of Health required; online regulated	2024: repeal of generational law; stricter product notification
France	Public Health Code (TPD); disposables banned (Law 2025 − 175)	Regulated as tobacco; stricter smoke-free spaces (Décret 2025 − 582)	Total ban (Decree TRIS 2025/0110/FR), suspended until Aug 2025	18+	Distance/online sales restricted	2025: disposable ban; expanded youth protection rules
Germany	TabakerzG (2016, TPD transposition), warnings, restrictions	Same as conventional tobacco	Classified as unauthorized novel foods, sale prohibited	18+ (JuSchG)	Allowed under national law	Novel food classification reaffirmed by BfR and courts
Spain	TPD-based rules; excise tax on e-liquids since 2025	EU flavor ban transposed (RD 47/2024)	Excise tax (2025); draft RD (TRIS 2025/0044/ES) with nicotine cap 0.99 mg/pouch (de facto ban)	18+	Allowed; taxed	2025: excise duty; draft decree on pouches
Italy	Legislative Decree 6/2016 (TPD-based); age checks, warnings	Treated as tobacco; favorable tax regime	Law 15/2022 and Decree 504/1995: notification and excise tax, not under TPD	18+; seller must verify	Online sales of nicotine products have been banned since 2018.	From 2023: ADM excise regime for pouches

* ENDS = Electronic Nicotine Delivery Systems; HTPs = Heated Tobacco Products; FDA = U.S. Food and Drug Administration; PMTA = Premarket Tobacco Product Application; TRPR = Tobacco and Related Products Regulations; MHRA = Medicines and Healthcare products Regulatory Agency; TPD = Tobacco Products Directive; PACT Act = Prevent All Cigarette Trafficking Act; BfR = Bundesinstitut für Risikobewertung (German Federal Institute for Risk Assessment); JuSchG = Jugendschutzgesetz (German Youth Protection Act); ADM = Agenzia delle Dogane e dei Monopoli (Italian Customs and Monopolies Agency); RD = Royal Decree; TRIS = Technical Regulation Information System

As evidenced in a systematic review assessing the effectiveness of regulatory strategies aimed at preventing or reducing e-cigarette use among youth (ages 12–21) in high-income countries [71], the most promising strategies include flavor bans, sales licenses, warning labels, and taxation, while age restriction, although the most widely adopted, demonstrated variable results.

Calls for comprehensive reform include aligning vaping laws with those regulating tobacco products: banning flavored e-liquids, enforcing plain packaging, and restricting use in public areas. Stronger surveillance, tax policies, and educational campaigns are also vital. Given the increasing health evidence and the risk of creating a new generation of nicotine users, regulatory frameworks must evolve rapidly to prioritize youth protection while supporting harm reduction strategies for adult smokers.

Evidence indicates that combined regulations, particularly when implemented early and in coordination, are more effective than isolated measures. A comprehensive strategy, including flavor and marketing bans, taxation, strict age-verification, restrictions on use in public places to limit both direct use and secondhand exposure, plain packaging, and sales licensing, will reduce product appeal, accessibility, and involuntary inhalation. Such an integrated approach targeting price, availability, and desirability is essential to protect adolescents from nicotine initiation and to safeguard public health [72].

Taxation of e‑liquids, enforcement of minimum‑price policies, and mandatory special licenses for vaping retailers have proven highly effective at curbing youth access by making products less affordable and more difficult for minors to obtain [71]. Moreover, revenue generated from these measures can be reinvested in prevention programs, creating a sustainable funding stream to support educational and cessation initiatives.

Ongoing surveillance of adolescent nicotine uses and robust research into the long‑term health effects of ENDS, heated‑tobacco products, and oral pouches remains essential. Public‑health agencies across Europe should systematically collect age‑specific data on single and dual‑use patterns and allocate funding to studies examining neurodevelopmental impact, respiratory outcomes, and addiction trajectories in youth, where current evidence is still limited.

Table 2 summarizes key policy recommendations and their intended targets for preventing youth nicotine use and safeguarding adolescent health.

**Table 2:** Key recommendations

Recommendation	Target
• *Enact and enforce comprehensive laws on age limits*,* advertising*,* packaging*,* clear labelling and taxation.* • *Ban all characterizing sweet and flavored additives.* • *Require strict licensing for vape retailers.* • *Ban marketing and sponsorship of ENDS/HTPs*,* aligning with existing restrictions on traditional cigarettes.* • *Extend plain packaging and graphic warnings to novel products.*	Policymakers, Regulators
• *Implement evidence-based*,* interactive school programs and curricula on nicotine and ENDS harm.* • *Use digital platforms*,* social networks*,* and peer-led programs to disseminate prevention messages.*	Educators, Schools
• *Mobilize parents and young community leaders (e.g.*,* “influencers”) in anti-vaping campaigns.* • *Promote smoke-free and vape-free homes.* • *Develop peer-to-peer support networks.* • *Conduct anti-vaping campaigns leveraging the widespread appeal of social media among young people*,* through collaborations with leading youth figures and content creators*	Community, Parents
• *Train pediatricians and clinicians to screen for smoking/vaping*,* counsel youth (and parents)*,* and refer to cessation resources.* • *Ensure accessible cessation programs for teens.* • *Launch mass-media and social media campaigns highlighting the dangers of all nicotine products and debunking myths.* • *Engage youth “influencers” for counter-messaging*	Public Health, Pediatricians
• *Fund continuous monitoring of youth nicotine use (surveys*,* clinical data).* • *Support research on long-term health effects of vaping and dual use in adolescents.*	University, Researchers

## Conclusion

The rise of e-cigarettes, HTPs, and nicotine pouches among adolescents represents a serious threat to public health, driven by misperceptions of safety, flavor-enhanced appeal, and pervasive marketing. Without decisive action, these novel products risk undermining decades of progress in reducing youth tobacco use. Policymakers must tighten regulations, banning flavored products, enforcing plain packaging, restricting online and social-media content, strengthening age-verification measures, and taxation. Schools and communities should implement interactive, evidence-based education programs and campaigns that prevent adolescents from using these products. Pediatricians play a role by routinely screening for all nicotine product use, delivering clear, age-appropriate counseling, and referring young patients to cessation resources. Continuous surveillance is essential to ensure that interventions remain effective and responsive to emerging trends. Only through sustained policy reform, collaboration, and clinical engagement can we protect the next generations from lifelong nicotine dependence and detrimental health effects.

## Data Availability

Not applicable.

## Abbreviations

e-cigarettes: electronic cigarettes
ENDS: Electronic Nicotine Delivery Systems
ESPAD: European School Survey Project on Alcohol and Other Drugs
FDA: Food and Drug Administration
HTPs: Heated Tobacco Products
SIMRI: Italian Pediatric Respiratory Society
SIP: Italian Society of Pediatrics
